# Correlation of CD4+ count and viral load with urinary tract infection and antimicrobial resistance pattern of bacterial uropathogens among HIV patients in Wolaita Sodo, South Ethiopia

**DOI:** 10.3389/fmicb.2024.1363287

**Published:** 2024-04-30

**Authors:** Admasu Haile Hantalo, Abera Kumalo Shano, Tekilu Israel Meja

**Affiliations:** ^1^School of Medical Laboratory Sciences, College of Medicine and Health Sciences, Wolkite University, Wolkite, Ethiopia; ^2^School of Medical Laboratory Sciences, College Health Sciences and Medicine, Wolaita Sodo University, Wolaita Sodo, Ethiopia; ^3^Department of Medical Laboratory Sciences, College of Health Sciences, Ethiopian Police University, Addis Ababa, Ethiopia

**Keywords:** urinary tract infection, correlation, CD4 count, viral load, antimicrobial resistance, Ethiopia

## Abstract

**Background:**

The permanence of HIV patients in healthcare provision centers exposes their weak immunity to various nosocomial microorganisms that migrate into and out of the hospital environment. The incidence of bacterial infections, including urinary tract infection, was inversely correlated with CD4+ T cells. Urinary tract infection (UTI) is one of the clinical problems among HIV patients. There was scarcity of published data on the relationship between viral load, CD4+ level, and UTI. This study aimed to assess the relationship between viral load and CD4 with bacterial UTI among HIV patients.

**Methods:**

The cross-sectional study was conducted in the Wolaita Sodo Town Health Center ART clinic. The socio-demographic data were collected using a pre-designed questionnaire. Patients' charts were reviewed to collect the current CD4 and viral load. Urine specimens were inoculated on blood agar, cysteine lactose electrolyte deficient (CLED) agar, and MacConkey agar, and bacterial species were finally identified using various biochemical methods. Antimicrobial sensitivity testing was conducted using standard microbiological tests. Bivariate and multivariate analyses were employed to describe the association between pairs of variables and to examine the relationship between independent variables and dependent variables.

**Results:**

In this study, the overall prevalence of urinary tract infection (UTI) was 13.7%. *Escherichia coli, Staphylococcus aureus, Pseudomonas aeroginosa, Staphylococcus saprophyticus, Proteus mirabilis*, and *Klebsiella pneumoniae* were bacterial uropathogens detected in this study. *E.coli* (45.7%) was the predominant isolate followed by *S. aureus* (14.3%). Positive correlation between CD4+ count and urinary tract infection was detected and found statistically significant (*r* = 0.288 *p* > 0.01), whereas the viral load and urinary tract infection negatively correlated and showed statistically significant association (*p* < 0.01). The resistance rate of *E.coli* was 94%, 75%, and 69% to ciprofloxacin, norfloxacin, and cefepime, respectively. This study revealed that *E.coli* exhibited 94% and 75% resistance to amoxicillin-clavulanic acid and tetracycline, respectively. *K. pneumoniae* demonstrated complete resistance (100%) to amoxicillin-clavulanic acid, tetracycline, and trimethoprim-sulfamethoxazole, while showing 100% susceptibility to ciprofloxacin and nitrofurantoin. In the present study, the magnitude of the multi-drug resistance (MDR) was found to be 80%. CD4+ count, combination of antiretroviral therapy (ART) drugs, and a history of hospitalization were risk factors for urinary tract infection.

**Conclusion:**

In the current study, urinary tract infection emerged as a significant health concern among people living with HIV following their ART. The occurrence of urinary tract infection among HIV patients could be influenced by multifactorial factors that require further study. The CD4+ count was positively correlated with the prevalence of UTI, whereas the viral load was negatively correlated. The CD4+ count, combination of ART, and history of hospitalization were independent risk factors for UTI. The prevalence of MDR bacterial pathogens were notably high. Therefore, the treatment of UTI in HIV patients should be prescribed based on antibacterial susceptibility testing results.

## Introduction

The destruction of CD4+ cells caused by human immunodeficiency virus (HIV) leads to acquired immunodeficiency syndrome (AIDS), resulting in progressive weakening of the immune system leading to the development of serious opportunistic infections (Schönwald et al., 1999). HIV, the virus with unique pathogenesis involving the gradual decline of immunity, reduces body's ability to fight off invading commensal organisms in people living with HIV (PLHIV) (Debalke et al., 2014; Skrzat-Klapaczyńska et al., 2018; Adhanom et al., 2019). HIV patients with low CD4 counts due to advanced AIDS are at the risk of developing renal syndromes and neurological complications such as hyperreflexia and hyporeflexia, which can lead to urinary stasis and ultimately infection (Rashmi et al., 2013). Urinary tract infection (UTI), which could be symptomatic or asymptomatic, is a notable opportunistic infection among HIV patients. People living with HIV (PLHIV) are at the high risk of developing UTIs that can progress eventually to bacteremia, sepsis, and potentially lead to death due to weakened immunity (Ifeanyichukwu et al., 2013; Barnie et al., 2019). Bacterial UTIs are more common and can progress to severe form under certain underlying conditions in individuals with HIV (Olowe et al., 2015). The permanence of HIV patients in hospitals increases the risk of nosocomial transmission of virulent microorganisms, which can migrate into and out of the hospital environment (Panis et al., 2009). *E.coli, K. pneumoniae, P. mirabilis, P. aeruginosa, S. aureus*, and *S. saprophyticus* are the most common bacterial etiologies of UTI (Panis et al., 2009; Marami et al., 2019; Simeneh et al., 2022).

Expanding resistance to available antibiotics used to treat UTIs is another concern, which may contribute to the emergence of MDR bacterial uropathogen strains in HIV patients (Debalke et al., 2014; Fenta et al., 2016). The burden associated with MDR pathogens is high in developing countries due to lack of advanced microbiological techniques, resulting in incorrect diagnosis, irrational use of antibiotics, and inadequate infection prevention strategies (Murugesh et al., 2014; Tuem et al., 2019; Simeneh et al., 2022). UTI is one of the public healthcare problems that decreases the quality of life and leads to work absence. Various studies conducted in Ethiopia, including those involving HIV patients, have reported prevalence rates ranging from 4% to 25% (Debalke et al., 2014; Fenta et al., 2016; Getu et al., 2017; Ayelign et al., 2018; Marami et al., 2019). Due to antimicrobial-resistant uropathogens, UTI is continuing as public health challenge, indicating that further research in this area is required with an objective to develop effective control mechanism of antibiotics usage and enhance infection prevention programs (Ifeanyichukwu et al., 2013; Fenta et al., 2016). The incidence of UTIs among HIV patients is higher in individuals with CD4 counts of <500cells/mm^3^, indicating an association between CD4 counts and bacterial UTI (Fenta et al., 2016). In Africa, it has also been revealed that PLHIV with CD4 cell counts <200 cells/mm^3^ are more likely to develop UTIs (Chaula et al., 2017; Tessema et al., 2020). There is scarcity of available data on correlation of bacterial UTI with CD4 cell counts, viral loads, and other predictive factors in the study area. Therefore, the current study aimed to detect antimicrobial-resistant bacterial uropathogens among PLHIV and their correlation with viral load and CD4 counts among HIV patients.

## Methods

### Study area

Health institution-based cross-sectional study was conducted in Wolaita Sodo Town Health Center (STHC) located in Wolaita Sodo town, South Ethiopia. STHC provides ART services for the Sodo town and neighboring woredas. The town has a latitude and longitude of 6°54′N 37°45′E with an elevation between 1600 and 2100 m above sea level. The mean annual temperature and rainfall of Wolaita Sodo ranges from 15.1 to 20°C and 1201–1600 mm, respectively. According Southern Nations, Nationalities and People Region bureau of Finance and Economic Development report, the town has a total population of 2,542,595, of whom 125,855 are men and 128,440 women. Wolaita Sodo town has three health centers (Wadu Health Center, Arada Health Center, and Sodo Town Health Center); among them, only STHC provides ART services. The total number of for were 650 in STHC provides ART follow-up for a total of 650 HIV-infected patients. Every working day, individuals living with HIV attend the ART Clinic for their regular follow-up.

### Study design

An institutional-based cross-sectional study was conducted among people living with HIV who were receiving healthcare at Wolaita Sodo town health centers from April to July 2021.

### Source population

The source population included all HIV-positive adults attending the WSHC ART clinic.

### Study population

The study population included all HIV-positive adults attending the ART clinic during the study period.

### Inclusion criteria and exclusion criteria

Adult patients who were not taking antibiotics for a current UTI were included in the study, while critically ill patients and those receiving any antibiotics treatment were excluded from the study.

### Sample size and sampling technique

The required sample size was determined using a single population formula, considering the following assumptions:

Prevalence = 11.3% prevalence of culture proven UTI among HIV patients in Ethiopia (Fenta et al., 2016)


$$\text{Sample size }({\text{n}}_{\text{o}})\text{=}\left(\text{z}\frac{\alpha }{\text{2}}\right)\text{2}.\text{p}(1\;-\text{p}){\text{/d}}^{\text{2}}$$

where

*n*_o_ = the required sample size, Z = Z score for 95 % confidence interval = 1.96,

*p* = prevalence; 11.3% (0.113), *d* = tolerable error =3 %.

1-*p* = *Q* = negative proportion.

Hence, *n* = [(1.96)2 × 0.113 × (1–0.113)]/(0.03)2 = 423.

If the total population was <10,000, then the following correction formula was applied:


$$\frac{\text{n}}{\text{1+}\frac{\text{n}}{\text{N}}}\text{=}\frac{\text{423}}{\text{1+}\frac{\text{423}}{\text{650}}}\text{=256},\text{ final sample size became }256.$$

The study participants were selected using the systematic sampling technique, with a predetermined Kth value, where Kth value = total population/sample size = 650/256 = 3. After randomly selecting the first participant among the first three early arrivals at the ART clinic, subsequent participants were selected at the intervals of three individuals until the required sample size was reached.

### Data collection and laboratory processing

The structured questionnaire was employed to collect socio-demographic characteristics and associated factors. Secondary data regarding ART and clinical history were retrieved from patient's charts. Participants' current CD4 value and viral load were collected from their medical records.

### Sample collection, handling, and transport

A sterile screw-capped container was used to collect mid-stream urine (MSU) samples. All urine specimens were promptly placed in a cold box and transported to the Central laboratory within 30 min of collection (Cheesbrough, 2005).

### Culture and identification

Cysteine lactose electrolyte deficient (CLED), medium, blood agar, MacConkey agar (MAC), and mannitol salt agar (MSA) were used to culture urine samples. A colony count ≥10^5^CFU/ml of urine was defined as a positive urine culture. All positive cultures were further identified by their colony characteristics. Gram staining was conducted to differentiate between gram positives and gram negatives. Pure isolate was sub-cultured onto a nutrient broth medium and incubated aerobically at 37°C for 12–24 h for biochemical testing (Cheesbrough, 2009). Additionally, a single isolated bacterium was inoculated onto a nutrient agar slant and stored in a refrigerator following 24 h of incubation for maintenance. A series of biochemical tests, including Kligler iron agar, Simmons citrate agar, lysine iron agar, urea, glucose, and lactose fermentation, lysine decarboxylation, gas and H_2_S production, motility tests, and indole (Cheesbrough, 2009), were used to identify gram-negative bacteria. Gram staining, catalase, coagulase, and novobiocin were used to identify gram-positive bacteria.

### Antimicrobial susceptibility testing

Antimicrobial susceptibility testing was conducted using the Kirby-Bauer disk diffusion method on Mueller–Hinton agar plates (Oxoid Ltd.) prepared to a 4 mm thickness. Selected antimicrobial disks, including ampicillin (10 μg), amoxicillin-clavulanic acid (20/10 μg), cefoxitin (30 μg), ciprofloxacin (5 μg), gentamicin (10 μg), tetracycline (10 μg), norfloxacin (10 μg), cefepime (30 μg), ceftriaxone (30 μg), nitrofurantoin (F) 300 μg, amikacin (10 μg), and azithromycin (30 μg) (Oxoid Ltd.), were used for susceptibility testing. After measuring the zone of inhibition diameter of bacterial isolates, it was compared with interpretative standards as in the CLSI guideline to classify as sensitive (S), intermediate (I), or resistance (R) (Sahu et al., 2018).

### Data processing and data analysis

The data were coded and entered into SPSS version 24 for analysis. After cleaning the data were cleaned using frequencies and cross-tabulation before regression analysis. Results were presented using tables, figures and charts after analysis of descriptive statistics and presented for continuous and discrete data in terms of mean and standard deviation for those normally distributed data. The binary logistic regression model was used to test the association between dependent and independent variables. All variables with a *p* < 0.25 in the binary regression analysis were included in the multivariable regression analysis. The degree of the association was interpreted using the adjusted odds ratio with 95% confidence intervals, and the significance level was declared at a *p* < 0.05.

## Results

### Socio-demographic characteristics

The data were collected from a total of 255 study participants; among them, 153 (60%) were female individuals and 102 (40%) were male individuals. Of the 255 total study participants, 43.9% belonged to the 28–37 age group. In terms of marital status, ~123 (43.9%) participants were married, and 77.25% of study participants were urban residents (Table 1).

**Table 1:** Bacteriuria and socio-demographic characteristics among ART followers in Wolaita Sodo, Ethiopia, 2021.

**Variables**	**Total**	**Bacterial growth**	
		**Yes (%)**	**No (%)**
**Sex**			
Male	102	14 (13.7)	88 (86.3)
Female	153	21 (13.7)	132 (86.3)
**Age (in year)**			
18–27	54	8 (14.8)	46 (85.2)
28–37	112	11 (9.8)	101 (90.2)
38–47	62	11 (17.7)	51 (82.3)
≥48	27	5 (18.5)	22 (81.5)
**Educational level**			
Illiterate	76	5 (6.6)	71 (93.4)
1–8	123	21 (17.1)	102 (82.9)
9–12	40	7 (17.5)	33 (82.5)
Diploma and above	16	2 (12.5)	14 (87.5)
**Marital status**			
Single	49	6 (12.2)	43 (87.8)
Married	123	13 (10.6)	110 (89.4)
Divorced	60	12 (20.0)	48 (80.0)
Widowed	23	4 (17.4)	19 (82.6)
**Residence**			
Urban	197	28 (14.2)	169 (85.8)
Rural	58	7 (12.1)	51 (87.9)

### ART and clinical related characteristics

Out of the total 255 study participants, 48.2% of the total study participants had been HIV-positive for 5 years and above and 38.4% of those had following their therapy for 2–5 years. The majority of the study participants (82.4%) had CD4 count value of > 200 cell/mm^3^. Among the total 255 study participants, 74.1% of study participants had <1.7log current viral load. TDF-3TC-EFV (60%) and AZT-3TC-NVP (26.7%) were the most common ART combinations used to treat HIV patients (Table 2).

**Table 2:** Bacteriuria and clinical characteristics among ART followers in Wolaita Sodo, Ethiopia, 2021.

**Clinical characteristics**	**Total**	**Urine culture result**	

		**Bacterial growth**	**No bacterial growth**
**Time of infection (in year)**			
<1	36	5 (13.9)	31 (86.1)
1–2	34	2 (5.9)	32 (94.1)
3–5	62	11 (17.7)	51 (82.3)
>5	123	17 (13.8)	106 (86.2)
**Period of ART (in year)**			
<1	53	7 (13.2)	46 (86.8)
1–2	68	8 (11.8)	60 (88.2)
2–5	98	15 (15.3)	83 (84.7)
>5	36	5 (13.9)	31 (86.1)
**Current viral load (in log scale)**			
<1.7log	188	22 (11.7)	166 (88.3)
<3log	35	3 (8.6)	32 (91.4)
>3log	32	10 (31.2)	22 (68.8)
**CD4 level/mm** ^3^			
<200	51	15 (29.4)	36 (70.6)
>200	204	20 (9.8)	184 (90.2)
**Current class of ART drug**			
First line	253	35 (13.8)	218 (86.2)
Second line	2	0 (0.0)	2 (100)
**Combination of ART drug**			
AZT-3TC-NVP	68	15 (22.1)	53 (77.9)
AZT-3TC-EFV	11	2 (18.2)	9 (81.8)
TDF-3TC-EFV	153	12 (7.8)	141 (92.2)
TDF-3TC-NVP	23	6 (26.1)	17 (73.9)
**Level of adherence**			
Good	172	19 (11.0)	153 (89.0)
Poor	83	16 (19.3)	67 (80.7)
**WHO clinical stage**			
I	134	10 (7.5)	124 (92.5)
II	82	14 (17.1)	68 (82.9)
III	39	11 (28.2)	28 (71.8)
**TB co-morbidity**			
Yes	20	3 (15.0)	17 (85.0)
No	235	32 (13.6)	203 (86.4)
**History of UTI**			
Yes	93	18 (19.4)	75 (80.6)
No	162	17 (10.5)	145 (89.5)
**History catheter usage**			
Yes	7	3 (42.9)	4 (57.1)
No	248	32 (12.9)	216 (87.1)
**History of hospitalization**			
Yes	20	11 (55.0)	9 (45.0)
No	235	24 (10.2)	211 (89.8)

### Prevalence of urinary tract infection

**Figure 1:**
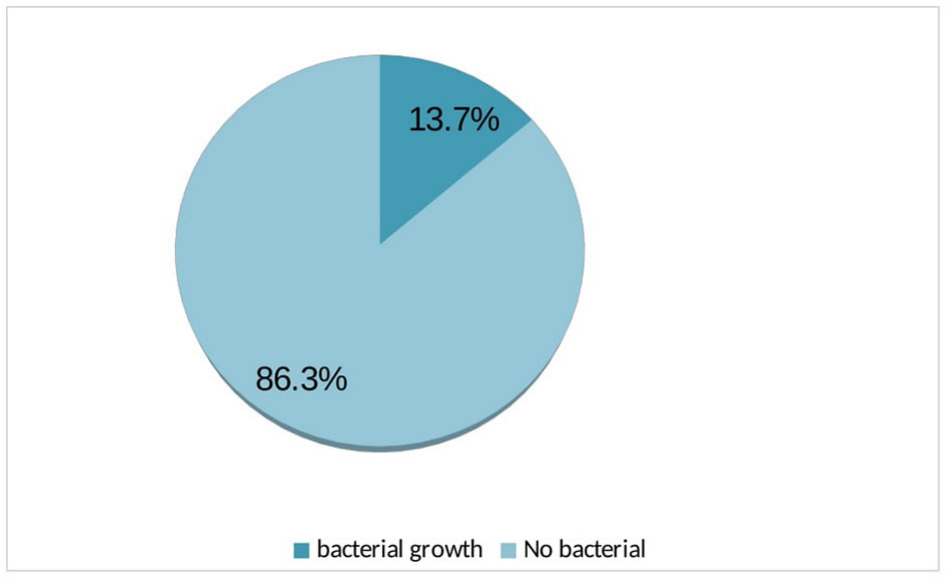
Bacterial growth among ART followers in Wolaita Soda, Ethiopia, 2021.

Urine cultures from 35 participants exhibited bacterial growth, resulting in the overall prevalence of 13.7% (Figure 1).

### Urinary tract infection with socio-demographic characteristics

The bacterial detection rate was similar in both sexes; however, among the total positive cases, female participants were more likely to be infected by UTI (Table 1).

### Bacterial etiologies of UTI

**Figure 2:**
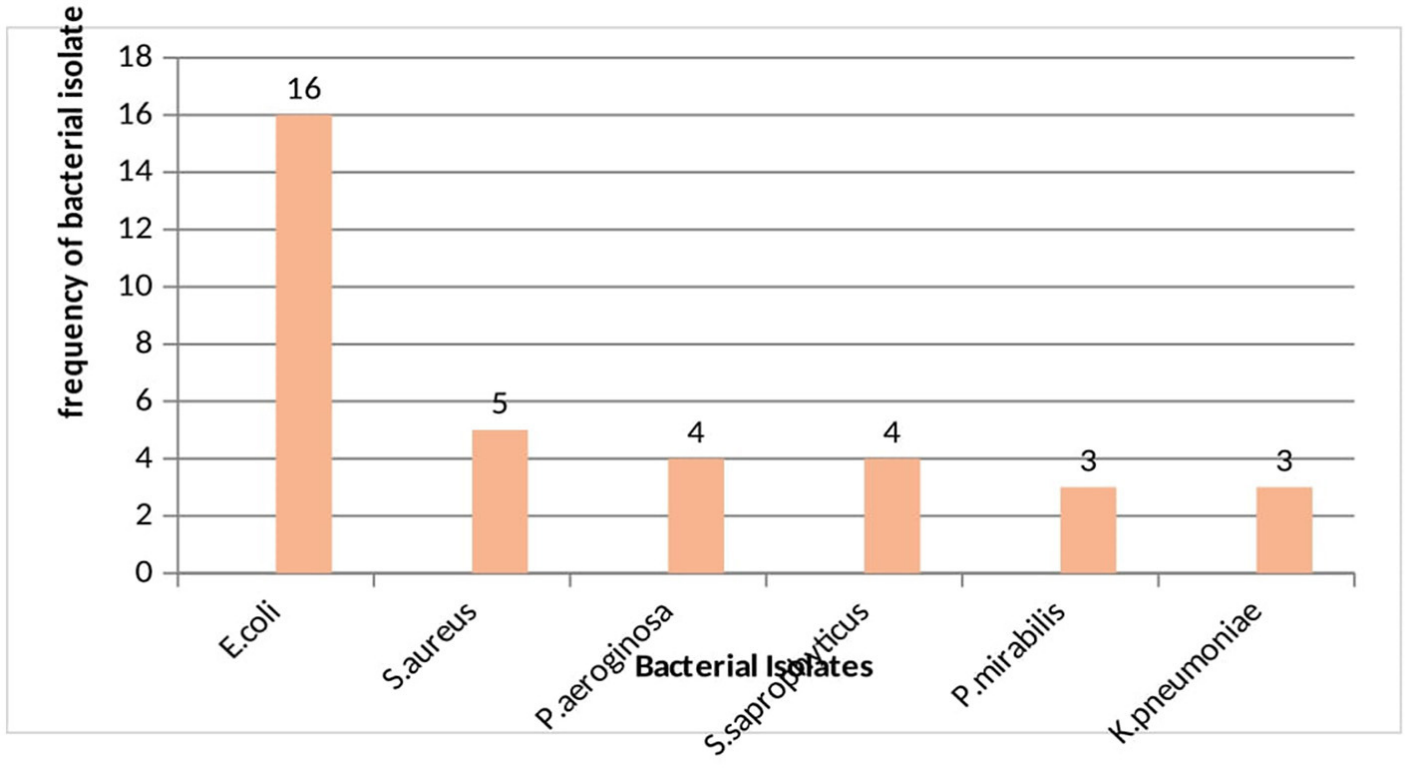
Distribution of bacterial isolates among ART followers in Wolaita Soda, Ethiopia, 2021.

Among the total of 35 uropathogenic bacterial isolates, 74.3% were gram negative bacteria and *E. coli* became the predominant isolate (45.7%) followed by *S. aureus* (14.3%) (Figure 2).

### Correlation of UTI with CD4 counts and viral load

As determined using Pearson's product correlation analysis, CD4 and bacterial growth were found to be low positive and statistically significant (*r* = 0.288 *p* > 0.01). This result showed that an increase in CD4 could lead to an increase in the prevalence of bacterial UTI in people living with HIV. Whereas, the viral load and bacterial growth negatively correlated and statistically significant (*r* = −0.153 *p* > 0.05). Pearson's product correlation analysis revealed a positive association between viral load and duration of ART with correlation coefficient (*r* = 0.262) and statistically significant (*p* < 0.01) as shown in the Table 3.

**Table 3:** Correlation analysis of UTI, CD4+ count, and viral load among ART followers in Wolaita Sodo, Ethiopia, 2021.

	**Time of ART**	**CD4 level/mm^3^**	**Current VL log scale**	**Bacterial growth**
Time of ART	1			
CD4 level/mm^3^	0.014	1		
VL log scale	0.263^**^	-0.410^**^	1	
Bacterial growth	-0.023	0.228^**^	-0.153^*^	1

^*^Correlation is significant at the 0.05 level (two-tailed). ^**^Correlation is significant at the 0.01 level (two-tailed).

### Predictive factors of urinary tract infections

The bivariate regression analysis was initially conducted, and variables with a *p* ≥0.25 were transferred to the multivariate regression analysis. CD4+ count [(AOR = 0.322, 95% CI = 0.108, 0.956)], current UTI [(AOR = 0.113, 95% CI = 0.041, 0.309)], and history of hospitalization [(AOR = 0.163, 95% CI = 0.041, 0.640)] were predictive factors of UTI. The likelihood of acquiring UTI in participants with high load was 2.147 times higher than in those with lower viral load (Table 4).

**Table 4:** Bivariate and multivariate analyses of risk factors for UTI among ART followers in Wolaita Sodo, Ethiopia, 2021.

**Variables**	**Bacterial growth**		**COR**	**p-value**	**AOR**	**P-value**

	**Yes**	**No**				
**CD4**+ **count**						
<200	15 (29.4)	36 (70.6)	1		1	
>200	20 (9.8**)**	184 (90.2)	3.833 (1.795-8.187)	0.001	0.322 (0.108-0.956)	0.041^**^
**Viral load**						
<1.7log	22 (11.7)	166 (88.3)	1		1	
<3log	3 (8.6)	32 (91.4)	3.430 (1.437-8.185)	0.005	0.769 (0.197-2.998)	0.705
>3log	10 (31.2)	22 (68.8)	4.848 (1.196-19.657)	0.039	2.147 (0.392-11769)	0.379
**Combination of ART**						
AZT-3TC-NVP	15 (22.1)	53 (77.9)	1		1	
AZT-3TC-EFV	2 (18.2)	9 (81.8)	1.247 (0.418-3.720)	0.692	1.184 (0.314-4.466)	0.803
TDF-3TC-EFV	12 (7.8)	141 (92.2)	1.588 (0.264-9.536)	0.613	1.344 (0.170-10.651)	0.780
TDF-3TC-NVP	6 (26.1)	17 (73.9)	4.147 (1.378-12.479)	0.011	4.892 (1.265-18.913)	0.021^**^
**WHO stage**						
I	10 (7.5)	124 (92.5)	1		1	
II	14 (17.1)	68 (82.9)	4.871 (1.885-12.590)	0.001^*^	2.613 (0.759-8.990)	0.128
II	11 (28.2)	28 (71.8)	1.908 (0.773-4.712)	0.161	1.161 (0.348-3.880)	0.808
**Symptom UTI**						
Yes	17 (50.0)	17 (50.0)	1		1	
No	18 (8.1)	203 (91.9)	11.278 (4.931-25.791)	0.000	0.113 (0.041-0.309)	0.000^**^
**History of hospitalization**						
Yes	11 (55.0)	9 (45.0)	1		1	
No	24 (10.2)	211 (89.8)	10.745 (4.045-28.542)	0.000	0.163 (0.041-0.640)	0.009^**^

^*^Statistical significant in multivariate regression only; ^**^statistically significant in both bivariate and multivariate regressions.

### Antimicrobial resistance profiles of uropathogenic bacteria: gram-negative bacteria

As shown in Table 5, antimicrobial resistance patterns of *E. coli* to amoxicillin-clavulanic acid, tetracycline, and trimethoprim-sulfomethoxazole were 94%, 75%, and 50%, respectively. *E. coli* showed susceptibility rates of 94%, 75%, 69%, 62.5%, 50% and 50% to ciprofloxacin, norfloxacin, cefepime, ceftriaxone, and trimethoprim-sulphamethazole (SXT), respectively. *P. aeruginosa* showed complete resistance (100%) to amoxicillin-clavulanic acid, cefepime, trimethoprim-sulphamethazole, and ciprofloxacin. *P. mirabilis* isolates were 60% sensitive to all antibiotics with complete sensitivity to norfloxacin, tetracycline, and ciprofloxacin. *K. pneumoniae* showed the higher rate of resistance to amoxicillin-clavulanic acid (100%), tetracycline (100%), and trimethoprim-sulphamethazole (100%). *K. pneumoniae* was 100% susceptible to nitrofurantoin, cefepime, and ciprofloxacin.

**Table 5:** Antimicrobial resistance profiles of gram-negative bacterial isolates (*n* = 26) ART followers in Wolaita Sodo, Ethiopia, 2021.

**Bacterial isolate**	**Pattern**	**Antibiotics**								
		**AMC**	**NOR**	**CTR**	**CFP**	**TC**	**SXT**	**NIT**	**AMK**	**CPR**
*E. coli* (*n =* 16)	S	0 (0)	12 (75%)	8 (50%)	10 (62.5 %)	2 (12.5%)	8 (50%)	11 (69%)	4 (25%)	15 (94%)
	I	1 (6%)	0 (0%)	4 (25%)	6 (37.5%)	2 (12.5%)	0 (0.0)	0 (0.0)	12 (75%)	1 (6%)
	R	15 (94%)	4 (25%)	4 (25%)	0 (0)	12 (75%)	8 (50%)	5 (31%)	0 (0)	0 (0.0)
*P. aeroginosa* (*n =* 4)	S	0 (0)	2 (50%)	1 (25%)	0 (0.0)	0 (0.0)	0 (0.0)	3 (75%)	1 (25%)	0 (0.0)
	I	0 (0)	0 (0.0)	0 (0.0)	0 (0.0)	2 (50%)	0 (0.0)	0 (0.0)	0 (0.0)	0 (0.0)
	R	4 (100)	2 (50%)	3 (75%)	4 (100%)	2 (50%)	4 (100%)	1 (25%)	3 (75%)	4 (100%)
*P. mirabilis* (*n =* 3)	S	2 (67%)	3 (100%)	2 (67%)	2 (67%)	3 (100%)	0 (0.0)	2 (67%)	2 (67%)	3 (100%0
	I	0 (0)	0 (0.0)	1 (33%)	1 (33%)	0 (0.0)	2 (67%)	1 (33%)	1 (33%)	0 (0.0)
	R	1 (33)	0 (0.0)	0 (0.0)	0 (0.0)	(0.0)	1 (33%)	0 (0.0)	0 (0.0)	0 (0.0)
*K. pneumoniae* (*n =* 3)	S	0 (0)	1 (33%)	3 (100%)	3 (100%)	0 (0.0)	0 (0.0)	2 (67%)	0 (0.0)	3 (100%)
	I	(0)	0 (0.0)	0 (0.0)	0 (0.0)	0 (0.0)	0 (0.0)	(0.0)	1 (33%)	0 (0.0)
	R	3 (100%)	2 (67%)	0 (0.0)	0 (0.0)	3 (100%)	3 (100%)	1 (33%)	2 (67%)	(0.0)

AMC, amoxicillin-clavulanic acid; NOR, norfloxacin; CTR, ceftriaxone; CFP, cefepime; TC, tetracycline; NIT, nitrofurantoin; AMK, amikacin; CPR, ciprofloxacin; SXT, trimethoprim-sulphamethazole.

### Gram-positive bacterial isolates

*S. aureus* exhibited susceptibility rates of 100%, 80%, 80%, and 60% to nitrofurantoin, ciprofloxacin, gentamicin, and azithromycin, respectively, whereas it showed 100%, 100%, and 80% resistance to ampicillin, cefoxitin, and tetracycline, respectively. *S. saprophyticus* showed complete resistance (100%) to ampicillin, amoxicillin-clavulanic acid, cefoxitin, and azithromycin, whereas it became 100% susceptible to gentamicin and nitrofurantoin (Table 6).

**Table 6:** Antimicrobial resistance profiles of gram-positive isolates (*n* = 9) among ART followers in Wolaita Sodo, Ethiopia, 2021.

**Bacterial isolate**	**Classification**	**Antibiotics**							
		**AMP**	**AMC**	**CXT**	**TC**	**AZM**	**GEN**	**NIT**	**CPR**
*S. aureus* (*n =* 5)	S	0 (0.0)	1 (20%)	0 (0.0)	1 (20%)	4 (60%)	4 (80%)	4 (100%)	4 (80%)
	I	0 (0.0)	2 (40%)	0 (0.0)	0 (0.0)	1 (40%)	1 (20%)	0 (0.0)	0 (0.0)
	R	5 (100%)	2 (40%)	5 (100%)	4 (80%)	0 (0.0)	0 (0.0)	1 (0.0)	1 (20%)
*S. saprophyticus* (*n =* 4)	S	1 (0.0)	0 (0.0)	0 (0.0)	2 (50%)	0 (0.0)	3 (100%)	4 (100%)	3 (75%)
	I	0 (0.0)	0 (0.0)	0 (0.0)	0 (0.0)	0 (0.0)	1 (0.0)	0 (0.0)	0 (0.0)
	R	3 (100%)	3 (100%)	4 (100%)	2 (50%)	4 (100%)	0 (0.0)	0 (0.0)	1 (25%)

AMP, Ampicillin; AMC, Amoxicillin-clavulanic acid; AZM, Azithromycin; CXT, Cefoxitin; GEN, Gentamicin; NIT, Nitrofurantoin; CPR, Ciprofloxacin; TC, tetracycline.

### Multiple drug resistance patterns of uropathogenic bacterial isolates

A total of 28 (80%) multi-drug-resistant (MDR) bacterial pathogens were detected from the total 35 bacterial isolates. As shown in Table 7, gram-negative bacteria accounts for 21 (75%) and gram positive 7 (25%). Among the gram-negative isolates, all *P. aeroginosa* and *K. pneumoniae* became MDR bacterial pathogens. Out of 28 MDR isolates, 15 (53.6%) were resistant to four classes of antibiotics.

**Table 7:** Multidrug resistance patterns of bacterial isolates among ART followers in Wolaita Sodo, Ethiopia, 2021.

**Bacterial isolates (MDR)**	**R3**	**R4**	**R5 and above**
**Gram negative**	7 (33.4%)	11 (52.4%)	3 (14.2%)
*E.coli* (*n =* 12)	6 (50%)	4 (33.4%)	2 (16.7)
*P. aeroginosa* (*n =* 4)	1 (25%)	3 (75%)	0 (0.0)
*P. mirabilis* (*n =* 2)	0 (0.0)	1 (50%)	1 (50%)
*K. pneumoniae* (*n =* 3)	0 (0.0)	3 (100%)	(0.0)
**Gram positive**	2 (25%)	5 (62.5)	1 (12.5%)
*S. aureus* (*n =* 4)	2 (50%)	2 (50%)	0 (0.0)
*S. saprophyticus* (*n =* 3)	0 (0.0)	2 (75%)	1 (25%)
*MDR*	9 (32.1%)	15 (53.6%)	4 (14.3%)

R3, resistant for 3 classes of antibiotics; R4, resistant for 4 classes of antibiotics; R5, resistant to 5 classes of antibiotics.

## Discussion

This study aimed at detecting bacterial UTI and antibacterial resistance and their correlation with CD4+ count and viral load among HIV patients. This study revealed that the overall culture-confirmed UTI prevalence was 13.7%. The finding was compared with previous studies conducted in Northeastern Ethiopia 11.6% (Alemu et al., 2020), Hawassa 13.6% (Nigussie and Amsalu, 2017), and Romania 12% (Chiţǎ et al., 2016). However, this finding was relatively lower than that of previous studies conducted in Addis Ababa 14.9% (Woldemariam et al., 2019), Gondar 17.8% (Yismaw et al., 2012), Nekemet 16.5%, and Sudan 19.5% (Hamdan et al., 2015). This finding was lower than that of other studies conducted in Nigeria that reported the range of UTI as 23.5–57.3% (Omoregie and Eghafona, 2009; Bigwan and Wakjissa, 2013; Kanu et al., 2016; Kemajou et al., 2016). This finding was relatively higher than that of other studies on UTI conducted in Addis Ababa 10.9% (Yeshitela et al., 2012) and Debre Tabor 10.9% (Worku et al., 2017). The variation might be due to by differences in socio-demographic characteristics, the host factor, and practices such as the social habits of the community, standards of personal hygiene, health education practices, and study population.

The study detected correlation between immune status and UTI. While many infections occur in immune-compromised people, UTIs are less common among this population compared to general population, with the exception of patients who have undergone renal transplantation (Hoepelman et al., 1992; Tolkoff-Rubin and Rubin, 1997). In current study, bacterial uropathogens were mainly isolated from HIV patients with a CD4 count of >200 cells/ml. This finding was corroborated with other study conducted in Chad (Abderrazzack et al., 2015). There was a significant relationship between CD4+ cell count and UTI (*p* < 0.05). This report was in line with other study conducted in the Netherlands to assess the relationship between bacteriuria and immune status (Hoepelman et al., 1992). In the current study, the prevalence of UTI was negatively correlated with viral load in HIV patients. Many studies reported numerous risk factors for UTI (Widmer et al., 2010; Ezechi et al., 2013; Skrzat-Klapaczyńska et al., 2018). In the present study, current CD4+ count, history of hospitalization, current UTI, and combination ART drugs were the predictive factors of UTI. This study demonstrated that 15 out of 35 participants with a CD4 count of <200/mm^3^ were infected by uropathogens. This finding was supported by other studies conducted in Tanzania (Chaula et al., 2017), India (Chandwani et al., 2022), and Brazil (Back-Brito et al., 2011). The most commonly used combination of ART drug was TDF-3TC-EFV to treat UTI. Bacterial growth was higher among TDF-3TC-NVP users (26.1%) followed by AZT-3TC-NVP users (22.1%). The bacterial growth was significantly higher among participants with high viral load (>3log) (31.5%). The bacterial profiling revealed that both gram-negative (*E. coli, K. pneumoniae, P. mirabilis* and *P. aeroginosa*) and gram-positive bacteria (*S. aureus* and *S. saprophyticus*) were involved. Gram negative bacteria (74.3%) were predominant causative agents of UTI. This is corroborated with other studies in which they were dominant bacterial uropathogens somewhere else including Ethiopia (Tolkoff-Rubin and Rubin, 1997; Omoregie and Eghafona, 2009; Bigwan and Wakjissa, 2013; Hamdan et al., 2015; Abate et al., 2017; Pragash et al., 2017).

In this study, *E. coli* became a predominant isolate (45.7%). This finding was in line with that of other studies conducted in Nigeria 50% (Ibadin et al., 2006). This finding was opposed with that of the study conducted in Nigeria, which reported that *S. aureus* was the most common uropathogen (Omoregie and Eghafona, 2009). Other study conducted in Nigeria (Kemajou et al., 2016) also reported comparable prevalence of bacterial pathogens, including *S. aureus* (29.7%), *E. coli* (28.5%), and *P. aeruginosa* (27.9%), which contradicted our study finding. This variation in bacterial etiology of UTI could be due to difference in pathogens from place to place, as well as variations in their susceptibility and resistance patterns. The criteria for choosing antibacterial agents should be based on the local availability of antibiotics and its expected susceptibility pattern to most likely pathogens because UTI are mostly treated empirically (Prakash and Saxena, 2013). Antimicrobial resistance pattern profiling was conducted on all isolated uropathogens using the Kirby-Bauer disk diffusion method, and the identified isolates showed varying patterns of antimicrobial resistance and susceptibility. A higher rate of resistance to amoxicillin-clavulanic acid, tetracycline, and trimethoprim-sulfamethoxazole was detected in gram-negative bacterial uropathogens, which was comparable with other studies conducted in Ethiopia that reported high resistance rate of antimicrobial agents (Kebamo et al., 2017; Nigussie and Amsalu, 2017). This aspect might be due to uncontrolled and easy access of antibiotics and overuse of the antimicrobial agents for long period. However, high susceptibility of gram-negative bacteria was detected to norfloxacin, ciprofloxacin, nitrofurantoin, and cefepime in the present study. According to the current study, the magnitude of MDR bacterial uropathogens was 80%. Comparable finding was reported from the previous study conducted in South Ethiopia 79.3% (Haile Hantalo et al., 2020) and Addis Ababa 78.4% (Fenta et al., 2016). This finding was absolutely higher than that of the study conducted in Ethiopia that reported 14.3% (Simeneh et al., 2022). However, this finding was relatively higher than that of other studies conducted in Ethiopia that revealed MDR rates of <50%−60% (Biset et al., 2020; Kasew et al., 2022). In contrast to our study, higher prevalence of MDR was reported from previous studies conducted in Gondar 91.7% (Yismaw et al., 2012) and north Ethiopia 95% (Alemu et al., 2012). This change in MDR patterns in the study area and across the world could be attributed to variations in antibiotic usage practices, including the prescription of antimicrobials, high frequency of antibiotic use, and lack of standard microbiological facilities.

## Conclusion

UTI is a significant cause of morbidity among HIV patients in the area. The CD4+ count was positively correlated with the prevalence of UTI, whereas viral load was negatively correlated. CD4+ count, combination of ART, current UTI, and history of hospitalization were independent risk factors for UTI. The prevalence of MDR bacterial pathogens was notably high. Therefore, the treatment of UTI in HIV patients should be prescribed based on antibacterial susceptibility testing results.

## Limitations of the study

Being a cross-sectional study, cause-effect relationship of CD4+ count and HIV load with bacterial UTI cannot be established. So, further study aimed at detecting cause-effect relationship of CD4+ cell count and HIV viral load and urinary tract infection is needed.

## Data availability statement

The raw data supporting the conclusions of this article will be made available by the authors, without undue reservation.

## Ethics statement

The studies involving humans were approved by the Wolaita Sodo University Research Ethical Committee. The studies were conducted in accordance with the local legislation and institutional requirements. The participants provided their written informed consent to participate in this study.

## Author contributions

AH: Conceptualization, Data curation, Formal analysis, Funding acquisition, Investigation, Methodology, Project administration, Resources, Software, Supervision, Validation, Visualization, Writing – original draft, Writing – review & editing. AS: Methodology, Supervision, Validation, Visualization, Writing – original draft, Writing – review & editing. TM: Formal analysis, Validation, Visualization, Writing – original draft, Writing – review & editing.
